# DNA methylation is associated with codon degeneracy in a species of bumblebee

**DOI:** 10.1038/s41437-023-00591-z

**Published:** 2023-01-19

**Authors:** H. Marshall, M. T. Nicholas, J. S. van Zweden, F. Wäckers, L. Ross, T. Wenseleers, E. B. Mallon

**Affiliations:** 1 Department of Genetics and Genome Biology, The University of Leicester, Leicester, UK; 2 Laboratory of Socioecology and Social Evolution, Department of Biology, KU Leuven, Leuven, Belgium; 3 Biobest Belgium N.V., Westerlo, Belgium; 4 The Lancaster Environmental Centre, University of Lancaster, Lancaster, UK; 5 The Institute for Evolutionary Biology, University of Edinburgh, Edinburgh, UK

**Keywords:** Evolutionary genetics, Social evolution, DNA methylation

## Abstract

Social insects display extreme phenotypic differences between sexes and castes even though the underlying genome can be almost identical. Epigenetic processes have been proposed as a possible mechanism for mediating these phenotypic differences. Using whole genome bisulfite sequencing of queens, males, and reproductive female workers we have characterised the sex- and caste-specific methylome of the bumblebee *Bombus terrestris*. We have identified a potential role for DNA methylation in histone modification processes which may influence sex and caste phenotypic differences. We also find differentially methylated genes generally show low levels of DNA methylation which may suggest a separate function for lowly methylated genes in mediating transcriptional plasticity, unlike highly methylated genes which are usually involved in housekeeping functions. We also examined the relationship between the underlying genome and the methylome using whole genome re-sequencing of the same queens and males. We find DNA methylation is enriched at zero-fold degenerate sites. We suggest DNA methylation may be acting as a targeted mutagen at these sites, providing substrate for selection via non-synonymous changes in the underlying genome. However, we did not see any relationship between DNA methylation and rates of positive selection in our samples. In order to fully assess a possible role for DNA methylation in adaptive processes a specifically designed study using natural population data is needed.

## Introduction

In many organisms, the generation of sexual dimorphic traits is mediated via genetically different sex chromosomes. In mammals, this consists of the classic XY system and in birds, the heterogametic sex is switched, displaying a ZW system (Dean and Mank, 2014). However, the diversity of sex determination systems in insects is considerably more varied. Some species display a X0 system where two X chromosomes are present in females and only one in males (Pal and Vicoso, 2015). Others lack sex chromosomes completely, for example, the mealybug, *Planococcus citri*, has a paternal genome elimination system where one whole set of chromosomes is highly condensed in males but not females. A similar system is found in Hymenoptera, haplodiploidy, here males have only a maternally derived haploid genome, generated from an unfertilised egg, whereas females are sexually produced diploids (Normark, 2003).

In addition to sexual dimorphism, social Hymenoptera also display phenotypically different castes of a single-sex. The differences between castes can be extreme in terms of morphology and behaviour. Some species rely on genetic inherited differences to generate castes, whilst others generate castes through environmental cues (Matsuura et al. 2018). For the latter species, this means all phenotypic differences are already encoded within the same genome, which suggests a role for epigenetic mechanisms. However, the repertoire of molecular mechanisms which allow these species to generate phenotypically different sexes and castes from a single genome remain unknown.

Epigenetic mechanisms have been proposed as a possible method for caste determination in multiple social insect species (reviewed in Sieber et al. 2021). The majority of research in this area has focused on DNA methylation, which is the addition of a methyl group to a cytosine nucleotide. Insect DNA methylation, like mammalian DNA methylation, is generally found in a CpG context (referring to a cytosine base immediately followed by a guanine base) (Glastad et al. 2014). It is found at lower levels, with <1–14% of CpGs being methylated, compared to mammals where around 70% of CpG sites are methylated (Bewick et al. 2016, Feng et al. 2010). Additionally DNA methylation in insects is generally located in gene bodies and associated with more highly expressed genes, such as housekeeping genes (Elango et al. 2009, Foret et al. 2009, Provataris et al. 2018). The function of DNA methylation in insects is largely unknown and thought to be variable based on the range of overall levels between taxonomic orders (Provataris et al. 2018). It has, however, been associated with alternative splicing (Flores et al. 2012), transcription factor binding (Glastad et al. 2016), and nucleosome occupancy (Lewis et al. 2020). In Hymenoptera, DNA methylation has been associated with caste differences in various species (Amarasinghe et al. 2014, Bonasio et al. 2012, Glastad et al. 2016, Lyko et al. 2010). However, a causal link has yet to be established (Oldroyd and Yagound, 2021). Also, no association between the level of sociality of a species and the level of DNA methylation has been found (Glastad et al. 2017, Weiner et al. 2013).

Many of the studies exploring a role for DNA methylation in the generation of different phenotypes in social insects have focused on single-sex caste differences, although see Glastad et al. (2016). However, recently DNA methylation has been strongly implicated in the generation of sex differences in some hemipteran bugs (Bain et al. 2021, Mathers et al. 2019), with other related species showing minimum sex-specific DNA methylation (Yu et al. 2022). This highlights the importance of assessing individual species rather than relying on observations of related species.

Previous work in Hymenoptera has also highlighted a role for the underlying genome in determining DNA methylation profiles. Wang et al. (2016) found DNA methylation profiles of alleles in offspring from hybrid crosses of the parasitic wasp *Nasonia* were almost identical to the methylation profile of the parent’s allele. It has also been shown in honeybees that genes which are more variable in terms of DNA methylation show increased genetic variability (Yagound et al. 2019) and that DNA methylation marks are heritable (Yagound et al. 2020). These studies clearly suggest a link between the epigenome and genome in these species. However, the functional importance of genetically determined DNA methylation remains unknown.

The bumblebee, *Bombus terrestris*, provides an ideal system to assess a potential role for DNA methylation in the generation of sex- and caste-specific phenotypes, in addition to exploring the relationship between the methylome and genome. *B. terrestris* is an important pollinator species both economically and ecologically (Woodard et al. 2015). It displays primitive eusociality with colonies consisting of a single female queen, mated by a single male, and female worker daughters. Exhibiting haplodiploidy, male *B. terrestris* are haploid and female queens and workers are diploid. Importantly, genome-wide DNA methylation differences have been found between sterile and reproductive phenotypes of *B. terrestris* workers (Marshall et al. 2019), with experimental manipulation of DNA methylation resulting in a change in worker phenotype (Amarasinghe et al. 2014). This suggests a functional role for DNA methylation in the generation of phenotypic differences between genetically similar individuals within this species. However, the extent to which DNA methylation differs between sexes and female castes of *B. terrestris* remains unknown. Additionally, previous research has shown colony-specific profiles of DNA methylation in *B. terrestris*, indicating a potential role for genotype-driven DNA methylation profiles in this species (Marshall et al. 2019).

In order to determine DNA methylation differences between sexes and castes of *B. terrestris* we have generated whole genome bisulfite sequencing (WGBS) libraries of males, queens, and reproductive workers. We have examined the genome-wide methylation profiles of each sex and caste and determined significant differentially methylated genes between castes and sexes. We also carried out whole genome re-sequencing of the same queen and male individuals to determine to what extent the methylation profile of a gene is related to the underlying genotype.

## Methods

### Sample collection

Four colonies of *B. terrestris* were established by Biobest, Leuven. Two colonies were generated from crosses of a *Bombus terrestris audax* queen and a *Bombus terrestris dalmatinus* male and two colonies of were generated from parents of the opposite subspecies, increasing the genetic diversity within our samples. They were then housed at the University of Leuven and kept in 21 ^∘^C with red light conditions, they were fed *ad libitum* with pollen and a sugar syrup. Callow workers were tagged with numbered disks in order to determine age. Worker reproductive status was confirmed by ovary dissection, ovaries were scored on a 0-4 scale as in Duchateau and Velthuis (1988), entire bodies were then stored at −80 ^∘^C along with the original queen mothers and male fathers. Three reproductive workers, aged 16–17 days, were selected from queenless conditions from each of the four colonies (supplementary 1.0.0).

### DNA extraction and sequencing

Whole genome bisulfite sequencing was generated for the parents and offspring of each colony. DNA was extracted from whole heads of the mother and father of each colony as well as from 12 reproductive workers (three per colony) using the Qiagen DNeasy®Blood & Tissue Kit following the manufacturers protocol. Reproductive workers were chosen to reduce the variation between samples as sterile and reproductive workers show different DNA methylation profiles (Marshall et al. 2019). Each sample was treated with RNAse A. DNA from the three reproductive worker samples per colony was pooled in equal quantities to produce one representative offspring sample per colony. DNA quantity and quality were determined by Nanodrop and Qubit® fluorometers as well as via gel electrophoresis. Samples were sent to BGI Tech Solution Co., Ltd.(Hong Kong) for library preparation, bisulfite treatment, and sequencing. Paired-end libraries (2 x 150bp) were sequenced across two lanes of an Illumina HiSeq 4000 platform with 40% phiX inclusion. A 1% lambda DNA spike was included in all libraries in order to assess bisulfite conversion efficiency, as the lambda genome is known to be unmethylated.

Whole genome re-sequencing of the parents was also carried out. DNA was extracted from half of the thorax of each mother and father per colony following a custom protocol (https://github.com/agdelafilia/wet_lab/blob/master/gDNA_extraction_protocol.md). DNA quantity and quality were determined by Nanodrop and Qubit® fluorometers as well as via gel electrophoresis. Samples were sent to Novogene Co., Ltd. for library preparation and sequencing. Paired-end libraries (2 x 150bp) were sequenced on an Illumina HiSeq 4000 platform.

### Generation of N-masked genomes

Differential DNA methylation analyses carried out using samples with different genomic backgrounds, i.e., non-inbred lines, can suffer strongly from reference genome bias, influencing the final differential DNA methylation calls (Wulfridge et al. 2019). To account for this we used whole genome re-sequencing data from the parents to call SNPs and create N-masked genomes per replicate colony.

Whole genome re-sequencing data of the parents were checked using fastqc v.0.11.5 (Andrews, 2010) and aligned to the reference genome (Bter_1.0, Refseq accession no. GCF_000214255.1, (Sadd et al. 2015)) using bowtie2 v.2.2.6 (Langmead and Salzberg, 2013) in *–sensitive* mode (supplementary 1.0.1). Aligned reads were deduplicated using GATK v.3.6 (McKenna et al. 2010). SNPs were called using Freebayes v.0.9.21.7 (Garrison and Marth, 2012) which accounts for ploidy differences between males and females. SNPs were then filtered using VCFtools v.0.1.16 (Danecek et al. 2011) with the following options: *–max-alleles 2 –minQ 20 –min-meanDP 10 –recode –recode-INFO-all*. A custom script was then used to filter SNPs to keep only homozygous alternative SNPs which are unique to either the mother or father of each colony. We also removed C-T and T-C SNPs as these are indistinguishable from bisulfite-converted bases in WGBS. This left a mean of 365,372 SNPs per colony (supplementary 1.0.2). The parental SNPs were then used to create an N-masked genome for each colony (four total) using the BEDtools v.2.28.0 *maskfasta* command (Quinlan and Hall, 2010).

### Differential DNA methylation between castes and sexes

Whole genome bisulfite sequencing (WGBS) data of the parents and pooled worker offspring were checked using fastqc v.0.11.5 (Andrews, 2010) and poor quality bases were trimmed using cutadapt v.1.11 (Martin, 2011). Libraries were then aligned to the colony-specific N-masked genomes created above using Bismark v.0.16.1 (Krueger and Andrews, 2011) and bowtie2 v.2.2.6 (Langmead and Salzberg, 2013) with standard parameters (supplementary 1.0.1). Bismark was also used to extract methylation calls, carry out deduplication, and destrand CpG positions. Coverage outliers (above the 99.9th percentile) were removed along with bases covered by less than 10 reads. The methylation status of each CpG was then determined via a binomial model, where the success probability is the non-conversion rate determined from the lambda spike. CpGs were classed as methylated when the false-discovery rate corrected *p*-value < 0.05. CpG sites were then filtered to remove any site that did not return as methylated in at least one sample.

Differential methylation was assessed at the CpG level in pair-wise comparisons (queen-male, queen-worker, male-worker) using the R package methylKit v.1.16.1 (Akalin et al. 2012). A logistic regression model was applied to each comparison with Benjamini-Hochberg correction for multiple testing (Benjamini and Hochberg, 1995), with subspecies as a covariate within the model. For a CpG to be differentially methylated a minimum difference of at least 10% methylation and a q-value of <0.01 were required. Genes were determined as differentially methylated genes if they contained an exon with at least two differentially methylated CpGs and an overall weighted methylation (Schultz et al. 2012) difference across the exon of >15%, based on previous research (Bain et al. 2021, Marshall et al. 2019). Two CpGs were chosen based on Xu et al. (2021), they find the methylation of two CpGs is enough to promote gene transcription in *Bombyx mori* via the recruitment of histone modifications.

### Relationship between the genome and methylome

Using the SNPs called above we created alternate reference genomes for the males and queens using the *FastaAlternateReferenceMaker* command from GATK v4.1.9.0 (McKenna et al. 2010). As reference genomes are in a haploid state we only used the alternative homozygous SNPs called above to substitute into the alternative references per individual. We then identified the codon degeneracy of every CDS sequence in each queen and male individualised genome following Mongue et al. (2019). Briefly, all codons were labelled per CDS and each base within each codon was labelled between 0-4 depending on how many nucleotide substitutions would be synonymous. We then determined the proportion of zero-fold and four-fold degenerate sites which were classed as methylated (determined via the binomial test above). We also checked the methylation level of a gene against the pN/pS (non-synonymous polymorphisms to synonymous polymorphisms) ratio of that gene (calculated using all genomes in a custom R script) to see if DNA methylation is enriched/depleted in genes potentially under selection. Significant relationships were determined using linear models in R, with interaction effects tested via two-way ANOVAs and post-hoc testing carried out using the *glht* function in the multcomp v.1.4 package (Hothorn et al. 2008).

### Gene ontology enrichment

Gene ontology (GO) terms for *B. terrestris* were taken from a custom database made in Bebane et al. (2019). GO enrichment analysis was carried out using the hypergeometric test with Benjamini-Hochberg (Benjamini and Hochberg, 1995) multiple-testing correction, *q* < 0.05, implemented from the R package GOStats v2.56.0 (Falcon and Gentleman, 2007). GO terms from differentially methylated genes between sexes and castes and GO terms associated with highly methylated genes were tested against a GO term database made from the GO terms associated with all methylated genes. Genes were determined as methylated if they had a mean weighted methylation level greater than the bisulfite conversion error rate of 0.05 in either queens, males or workers. GO terms associated with hypermethylated genes in any given sex/caste were tested for enrichment against GO terms from the given comparisons differentially methylated genes. REVIGO (Supek et al. 2011) was used to generate GO descriptions from the GO ids.

## Results

### Genome-wide sex- and caste-specific DNA methylation

**Fig. 1:**
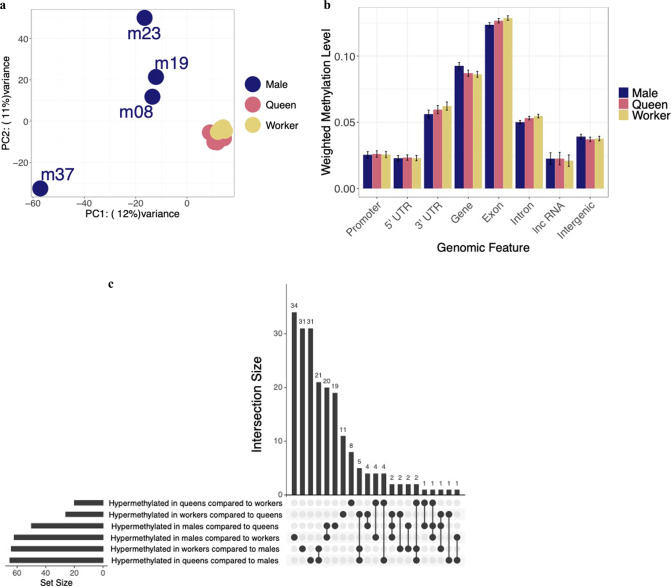
Caste- and sex-specific DNA methylation profiles. **a** PCA plot based on the methylation level per CpG for all CpGs which had greater than 10X in all samples and were classed as methylated in at least one sample (*n* = 5304). **b** Bar plot of the mean methylation level of each genomic feature for sexes and castes. Error bars represent 95% confidence intervals of the mean. Promoters are putative and represent 500bp upstream of a gene without any other genomic feature overlap. **c** Upset plot showing common genes containing a hypermethylated exon per hypermethylated sex/caste per comparison. The set size indicates the total number of hypermethylated genes, the intersection size shows how many of those are common between sets, as indicated by the connections in the bottom panel. E.g. 34 genes are uniquely hypermethylated in males compared to workers.

Here we examine the first genome-wide DNA methylation profiles of *B. terrestris* males and queens and compare these profiles to reproductive workers. We find low genome-wide DNA methylation levels in both sexes and castes, similar to those previously reported in workers by Bebane et al. (2019) and Marshall et al. (2019). On average 0.23% ± 0.05% of CpGs are methylated across all samples, with little overall variation in genome-wide levels between sexes and castes (supplementary 1.0.1). Reproductive workers, queens, and males do, however, show different CpG methylation profiles, with males showing more variation between samples and also clustering away from the two female castes (Fig. 1a). Additional variation is explained by individual samples rather than colony-level effects (supplementary Fig. S1).

Genome-wide, we see overall similar levels of DNA methylation across various genomic features for both sexes and castes (Fig. 1b). It has recently been shown that promoter DNA methylation exists in some insect species (Bain et al. 2021, Lewis et al. 2020). We have, therefore, annotated putative promoter regions in *B. terrestris*, defined at 500bp upstream of a gene with no overlap with other genomic features, we also added UTR regions and intergenic regions to further explore the genome-wide methylation profile. We chose 500bp for putative promoters in order to take a conservative approach. We have previously used 2000bp in another species (Bain et al. 2021) however, in *B. terrestris* we lost many putative promoter regions when using this size as they overlapped with other genomic features. We find the highest levels of DNA methylation for all sexes and castes are within exon regions, whilst putative promoter, and 5′ UTR regions show a depletion in DNA methylation compared to intergenic regions (Fig. 1b).

We also segregated genes into categories of differing levels of DNA methylation to explore the potential function of highly methylated genes across sexes and castes. There are a small number of genes classed as highly methylated across each sex/caste (supplementary Fig. S4, supplementary 1.0.3), weighed methylation level >0.7 as in previous work (Liu et al. 2018). Most highly methylated genes in queens and workers are also found in another caste/sex. Whereas males show a larger number of unique genes which are highly methylated (supplementary Fig. S4, *n* = 17). We then carried out an gene ontology enrichment test for each list of highly methylated genes per sex/caste and compared these to lists of genes classed as methylated (i.e., a weighted methylation level across the gene greater than the lambda conversion rate) for each sex/caste. We find a variety of GO terms enriched across sexes and castes mostly involved in core cellular processes (supplementary 1.0.4). Male highly methylated genes did, however, have a few GO terms enriched for eye pigmentation (GO:0008057, GO:0006726, GO:0048069) which were not present in the queen and worker enriched GO terms.

### Differential DNA methylation between sexes and castes

A differential DNA methylation analysis between sexes and castes found a total of 1232 differentially methylated CpGs between males and reproductive workers, 1034 differentially methylated CpGs between males and queens, and 358 differentially methylated CpGs between queens and reproductive workers. Roughly equal numbers were hypermethylated in each sex/caste per comparison, except for males and queens where queens show slightly more hypermethylated sites (Chi-squared Goodness of Fit: *χ*^2^ = 11.28, *df* = 1, *P* < 0.01, male *n* = 463, queen *n* = 571). The majority of all differentially methylated CpGs are located within genes and specifically within exons, we also find a slight depletion of differentially methylated CpGs in the first exon compared to the following exons (supplementary Fig. S2), this is in line with DNA methylation being slightly lower in the first exon in *B. terrestris* (Lewis et al. 2020).

We next classed a gene as differentially methylated if a given exon contained at least two differentially methylated CpGs and had an overall weighted methylation difference of at least 15%. We find 161 genes are differentially methylated between males and workers and males and queens (with some differences between the lists) and 59 between queens and workers (supplementary 1.0.5). Of these genes, around half have only one exon which is differentially methylated (supplementary Fig. S3). Of those with more than one differentially methylated exon, roughly equal numbers have exons showing the same direction of methylation difference and differing directions of methylation, i.e., some exons are hypermethylated and others hypomethylated in the same gene (supplementary Fig. S3). This may be suggestive of a role for DNA methylation in alternative splicing, as has been suggested previously in honeybees (Flores et al. 2012).

Previous research has also suggested there are two classes of methylated genes in arthropods, highly methylated genes involved in housekeeping functions and genes with lower levels of DNA methylation which are more plastic (e.g., Asselman et al. 2016). We find almost all genes classed as differentially methylated in this study do indeed show low levels of DNA methylation (>0 and <0.3) in all three castes, with a few showing medium levels (>=0.3 and <0.7) and none being classed as highly methylated genes in any sex/caste (supplementary Fig. S4).

We carried out a GO enrichment analysis on all differentially methylated genes and on hypermethylated genes for each sex/caste per comparison (supplementary 1.0.6). Whilst most terms are involved in core cellular processes, we specifically find differentially methylated genes between queens and workers are enriched for chromatin-related terms (e.g., “*histone H3-K27 acetylation*” (GO:0006338) and “*chromatin remodeling*” (GO:0097549)) and reproductive terms (e.g., “*germ cell development*” (GO:0007281) and “*sexual reproduction*” (GO:0019953)).

Differentially methylated genes between males and workers were also enriched for a large number of histone modification-related terms (e.g., “*regulation of histone H3-K9 methylation*” (GO:1900112), “*regulation of histone deacetylation*” (GO:0031063)) as well as reproductive related terms (e.g., “*gamete generation*” (GO:0007276), “*oviposition*” (GO:0018991) and “*spermatogenesis*” (GO:0007283)). Multiple histone-related terms and reproductive terms were also found for differentially methylated genes between males and queens, as well as the above we also found “*histone H4-K20 demethylation*” (GO:0035574), “*histone H3-K27 acetylation*” (GO:0043974), “*histone H3-K27 demethylation*” (GO:0071557) and “*positive regulation of histone H3-K9 trimethylation*” (GO:1900114). When looking specifically at hypermethylated genes per sex/caste compared to all differentially methylated genes per comparison we find various regulatory terms throughout, including terms involved in hormone regulation enriched in male hypermethylated genes compared to queens (supplementary 1.0.6).

Around 66% of all differentially methylated genes occur uniquely between comparisons, with 69/205 (~33%) genes occurring in multiple comparisons (Fig. 1c). Specifically, we find 21 genes are hypermethylated in queens and workers when compared to males and 20 genes are hypermethylated in males when compared to queens and workers. We carried out a GO enrichment on these genes using all differentially methylated genes from all comparisons as a background set. We find general cellular processes enriched in both gene lists with hypermethylated genes in the males compared to the female castes also enriched for hormone regulatory processes (e.g., “*regulation of hormone levels*” (GO:0010817) and “*hormone secretion*” (GO:0046879)).

### Relationship between the genome and the methylome

Recent research has indicated that the underlying genome may play a role in the establishment of DNA methylation profiles in various insect species (e.g., Wang et al. 2016, Yagound et al. 2020), including *B. terrestris* (Marshall et al. 2019). However, the function of gene-body DNA methylation in insects remains unknown (Provataris et al. 2018). It has been suggested that DNA methylation induced by the environment may either be a direct substrate for selection or itself induce genetic variation (via the deamination of cytosines to thymines), creating phenotypic variation (Skinner and Nilsson, 2021).

To explore the relationship between the genome and methylome in this context, we used whole genome re-sequencing data from the males and queens to see if DNA methylation appears to be directly associated with the underlying genome. We determined the proportion of zero-fold and four-fold degenerate sites which are classed as methylated (via the binomial test described above) to see if DNA methylation is enriched at sites which may be subject to selection (zero-fold sites, which always result in an amino acid change when there is a polymorphism) compared to sites protected from selection (four-fold sites, for which any polymorphism would result in the same amino acid).

**Fig. 2:**
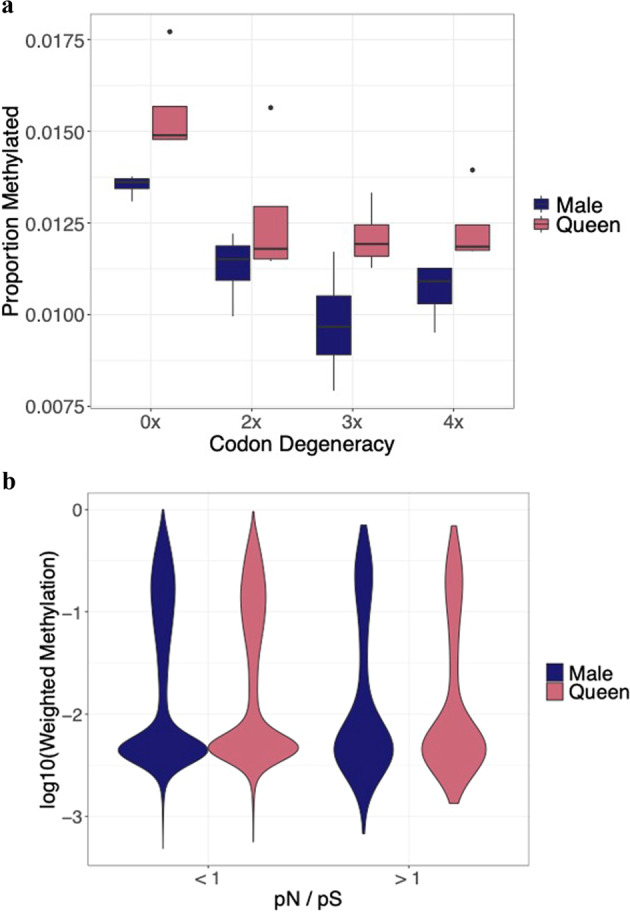
Relationship between DNA methylation and the underlying genome. **a** Boxplot showing the proportion of methylated sites per codon degeneracy level by sex. With zero-fold degenerate sites exposed to selection and four-fold degenerate sites shielded from selection. Outliers are show by black points. **b** Violin plot, showing data via a mirrored density plot, of the DNA methylation levels, by sex, of all genes with a pN/pS > 1 (i.e., genes under positive selection, *n* = 398) and a pN/pS < 1 (i.e. genes under stable selection, *n* = 14,934).

We find that a higher proportion of zero-fold degenerate sites are methylated compared to two-, three- and four-fold sites (beta regression: *z* = −126.4, *P* < 0.001, Fig. 2a), with queens showing a higher proportion of methylated sites across all degeneracy-levels compared to males. This higher methylation in queens compared to males across all sites is likely driven by queens generally having higher levels of DNA methylation in coding regions compared to males (Fig. 1b). It’s also worth noting that the zero-fold degenerate sites are only found in codon positions one and two, and so by association these positions have a higher proportion of methylated sites compared to the third codon position (supplementary 1.0.7). We also checked the overall methylation levels of genes which contain at least one methylated zero-fold degenerate site. Most genes with at least one methylated zero-fold degenerate site are common between males and queens (n = 341), with males having 26 unique genes containing a methylated zero-fold degenerate site and queens having 117. A gene ontology enrichment analysis of these genes revealed a large variety of processes, many of which were metabolic in nature (supplementary 1.0.8). Whilst most genes which contain a methylated zero-fold degenerate site are located in lowly methylated genes (Fig. S5), this is not a significant enrichment (hypergeometric test, for males: corrected *P* = 0.99, for queens: corrected *P* = 0.99). This is also the case in highly methylated genes (hypergeometric test, for males: corrected *P* = 0.43, for queens: corrected *P* = 0.21). However, we do find a significant enrichment of methylated zero-fold degenerate sites in genes with medium levels (weighed methylation >=0.3 and <0.7) of methylation (hypergeometric test, for males: corrected *P* < 0.001, for queens: corrected *P* = <0.001). We also checked to see if differentially methylated positions between males and queens are associated with a particular degeneracy level. We find most differentially methylated sites occur at zero-fold degenerate sites, although this mirrors the location of DNA methylation generally (supplementary Fig. S5).

Finally, we had enough alleles in our study (n = 12, four diploid queens and four haploid males) to carry out a pN/pS analysis to look for signatures of selection across the genome. We do not find any association with male or female levels of DNA methylation and potential selective pressure (linear model, *F*_1,15330_ = 1.449, *t* = −1.204, *P* = 0.228, Fig. 2b).

## Discussion

In this study we have explored the sex- and caste-specific DNA methylation profiles of *B. terrestris* and examined the relationship between the methylome and underlying genome. We find that sexes and castes show similar genome-wide DNA methylation profiles, with more variability in males. We also find there are a number of genes which are differentially methylated between sexes and castes, mostly between males and the female castes, involved in the regulation of histone modifications and reproductive-related processes. These differentially methylated genes show low levels of DNA methylation generally in all castes. Finally, we find that DNA methylation is enriched at sites exposed to selection in *B. terrestris*, i.e., zero-fold degenerate sites. However, we did not find an association between DNA methylation and genes potentially under selection.

### *B. terrestris* caste- and sex-specific methylome

Genome-wide we found that males show similar DNA methylation profiles to the two female castes, in terms of DNA methylation localisation to exons and depletion in promoter regions, we also found a number of differentially methylated genes between males and females. Previous research in other social insects has found variable results in terms of higher variability between sexes or castes. For example, the ants, *Solenopsis invicta* (Glastad et al. 2014), and *Camponotus florisanus* (Bonasio et al. 2012) show more variation between sexes compared to castes. Whereas, the ant *Harpegnathos saltator* (Bonasio et al. 2012) and the social termite *Zootermopsis nevadensis* (Glastad et al. 2016) show higher variability between castes compared to sexes. Additionally, the termite *Macrotermes natalensis* (Harrison et al. 2022) shows roughly equal variation between castes and sexes, highlighting the variability in DNA methylation differences within social insects.

Specifically, differentially methylated genes between male and female *B. terrestris* are enriched for many histone modification related processes. It has recently been found in the silk moth that the presence of DNA methylation promotes histone H3-K27 acetylation which changes the chromatin formation of a region allowing changes in gene expression (Xu et al. 2021). The relationship between DNA methylation and histone modifications in social insects remains unknown. However, recent work by Choppin et al. (2021) shows a role for histone acetylation in the regulation of worker reproduction and gene expression in the ant *Temnothorax rugatulus*. Additionally, Dixon and Matz (2022) find gene-body methylation across a variety of invertebrates, including *B. terrestris* is not sufficient to fully explain changes in gene transcription, they also suggest a more complex regulatory mechanism may be in play involving other epigenetic modifications. An exploration of the functional relationship between DNA methylation and histone modifications is needed across a greater diversity of insect species in order to understand how these processes may interact to produce downstream gene expression and thus phenotypic differences.

We also find differentially methylated genes between queens and reproductive workers are involved in reproductive related processes. Previous work has suggested a role for DNA methylation in reproduction in *B. terrestris* (Amarasinghe et al. 2014), as well as other social insects (Bonasio et al. 2012, Wang et al. 2020), although this does not appear to be consistent across Hymenoptera (Libbrecht et al. 2016, Patalano et al. 2015). Whilst the differentially methylated genes identified here suggest a role for DNA methylation in maintaining or generating sex and caste differences, a direct causal link between DNA methylation and the gene expression changes mediating phenotypes has yet to be found. The development of CRISPR-Cas9 DNA methylation editing (Vojta et al. 2016) in *B. terrestris* would allow for studies into the causal consequences of gene methylation.

Whilst the function of gene-body DNA methylation remains unknown, higher levels have been associated with stable gene expression of housekeeping genes (e.g., Provataris et al. 2018). Reciprocally, previous work suggested that low methylation of genes allows for more variation in gene expression levels increasing phenotypic plasticity (Roberts and Gavery, 2012). We find evidence in support of this idea in that all of the genes found to be differentially methylated between castes and sexes show overall low levels of DNA methylation, with no highly methylated genes differentially expressed. However, it is worth noting that there are proportionally more lowly methylated genes in this species compared to highly methylated genes which may also account for this trend. In addition, whilst the differential DNA methylation analysis done here is particularly stringent for this field, requiring a percentage difference in DNA methylation levels will allow for more lowly methylated genes to be identified, compared to highly methylated genes which would require overall greater differences in DNA methylation to meet the 15% threshold. Nevertheless, there is some mixed evidence across invertebrates that suggests lowly methylated genes respond to environmental changes as a group by increasing methylation levels and thus decreasing transcriptional levels, with the opposite occurring for highly methylated genes, providing transcriptional plasticity, known as the ‘seesaw’ hypothesis (Dixon and Matz, 2022, Dixon et al. 2018). A re-analysis of reproductive and sterile bumblebee worker WGBS data as part of Dixon and Matz (2022), found some support for this theory. A study specifically addressing this idea, which is able to disentangle tissue-specific profiles is needed. Additionally, we found many genes which are differentially methylated contain both hyper- and hypomethylated exons within the same gene. As discussed above, rather than directly regulating gene expression, this finding suggests an alternative role for DNA methylation, possibly in regulating alternative splicing, as has been shown previously in honeybees (Flores et al. 2012).

### DNA methylation and genome evolution

We next assessed the relationship between the underlying genome and the methylome. As discussed in Dixon and Matz (2022), gene-body DNA methylation does not appear to directly regulate gene expression in invertebrates. However, recent papers have suggested a role for DNA methylation in species evolution, whereby changes in DNA methylation in response to environmental conditions are directly heritable (Harney et al. 2022, Skinner and Nilsson, 2021) and may produce adaptive phenotypes. One mechanism which may allow this to occur is through DNA methylation-mediated genome mutation. The presence of DNA methylation has been shown, in humans, to increase mutation rates by over ten-fold compared to background genomic rates via the spontaneous deamination of cytosines (Sved and Bird, 1990). DNA methylation has also been shown to be linked to the underlying genotype in various insect species (Marshall et al. 2019, Wang et al. 2016, Wu et al. 2020, Yagound et al. 2019, 2020). If DNA methylation is acting as an environmentally-responsive targeted mutagen, we would expect to see it enriched in positions which would have an affect on gene expression levels or protein changes, i.e., where phenotypic changes would occur, providing substrate for selection.

In order to explore the above idea we examined the relationship between DNA methylation and codon degeneracy. We found that DNA methylation is enriched at zero-fold degenerate sites (i.e., those which produce an amino acid change if a substitution occurs). This trend is also observed in humans (Chuang and Chen, 2014). Chuang and Chen (2014) argue that enrichment of DNA methylation at zero-fold degenerate sites is not to act as a mutagen for such sites but rather to serve as a stabilising factor for these sites. The enrichment of DNA methylation in housekeeping genes in insects may be supportive of this idea. However, we find genes that contain methylated zero-fold degenerate sites in *B. terrestris* are mostly in lowly methylated genes, with enrichment of these sites in genes with mid-levels of methylation. This finding is suggestive of multiple roles for DNA methylation in insects, with highly expressed genes displaying high levels of potentially stabilising DNA methylation and other more plastic genes showing lower levels of DNA methylation. This is, however, purely a suggestion based on our results. We do not have gene expression data from these individuals to explore this further. Additionally, accurate annotation of housekeeping genes in *B. terrestris* is currently lacking. In order to test this idea, long term experimental evolution studies are needed to see if environmentally responsive DNA methylation does indeed drive genomic mutation and if this is targeted to highly or lowly methylated genes with or without housekeeping functions.

Finally, given the number of alleles available in our study we examined the association between DNA methylation and genome mutation further. We did not find any relationship between DNA methylation levels of a gene and the pN/pS ratio. This may be due to a number of reasons. Firstly, there may simply be no relationship between DNA methylation and rates of positive selection in *B. terrestris*, countering the idea that DNA methylation is providing substrate for selection. Secondly, the samples used in this study are from commercial colonies and as such may not be under any particular environmental pressures driving selection. Exploring this relationship in natural populations would provide a better indicator for a potential role of DNA methylation in adaptive processes. Additionally, Park et al. (2011) found the proxy for DNA methylation (CpG observed/expected density) was negatively correlated with rates of both synonymous and non-synonymous substitutions between species of the parasitic wasp *Nasonia*. Again, this supports the idea that methylation is present in constrained genes, potentially acting as a stabilising factor. However, as mentioned above this needs to be assessed fully in terms of highly methylated and lowly methylated genes rather than on a genome-wide scale in order to fully understand this relationship.

In summary, it is still unclear whether a potential function of gene-body DNA methylation is to act as a mutagen or stabilising factor in insects, with suggestive evidence available for both scenarios. A recent paper exploring epigenetic-mediated mutation in *Arabidopsis thaliana* also found evidence for both possibilities (Monroe et al. 2022). DNA methylation was found to increase rates of nucleotide substitution but was also associated with decreased rates of insertions and deletions, known to be more deleterious. This may help to explain how DNA methylation can act to maintain essential housekeeping gene function and also potentially provide targeted mutations within environmentally-responsive genes.

### Conclusion

We have characterised the sex- and caste-specific methylome of *B. terrestris* identifying a potential role for DNA methylation in downstream epigenetic regulatory processes which may influence sex and caste phenotypic differences. These results are correlational but can direct future experimental manipulation of DNA methylation profiles at specific genes. We also find that differentially methylated genes are those showing low overall levels, this may be due to the nature of the statistical analysis used to identify differential DNA methylation or it could suggest a function for low-levels of DNA methylation in mediating plasticity of gene expression. We also find that DNA methylation is enriched at zero-fold degenerate sites and suggest this may be explained by DNA methylation functioning as a mutagen at these sites to provide substrate for selection via changes in the underlying genotype.

## Data accessibility

Data has been deposited in GenBank under NCBI BioProject: PRJNA779586. All code is available at: https://github.com/MooHoll/Parent_of_Origin_Methylation.

## Supplementary information

Supplementary 1

Supplementary 2
