# Supplementary material for: DNA methylation is associated with codon degeneracy in a species of bumblebee
Source: Heredity (Edinb). 2023 Jan 19;130(4):188–95. doi: 10.1038/s41437-023-00591-z (PMC10076500; doi:10.1038/s41437-023-00591-z)
Supplement: Supplementary file 1 — Supplementary 1 [file 41437_2023_591_MOESM1_ESM.pdf]

Supplementary figures and text

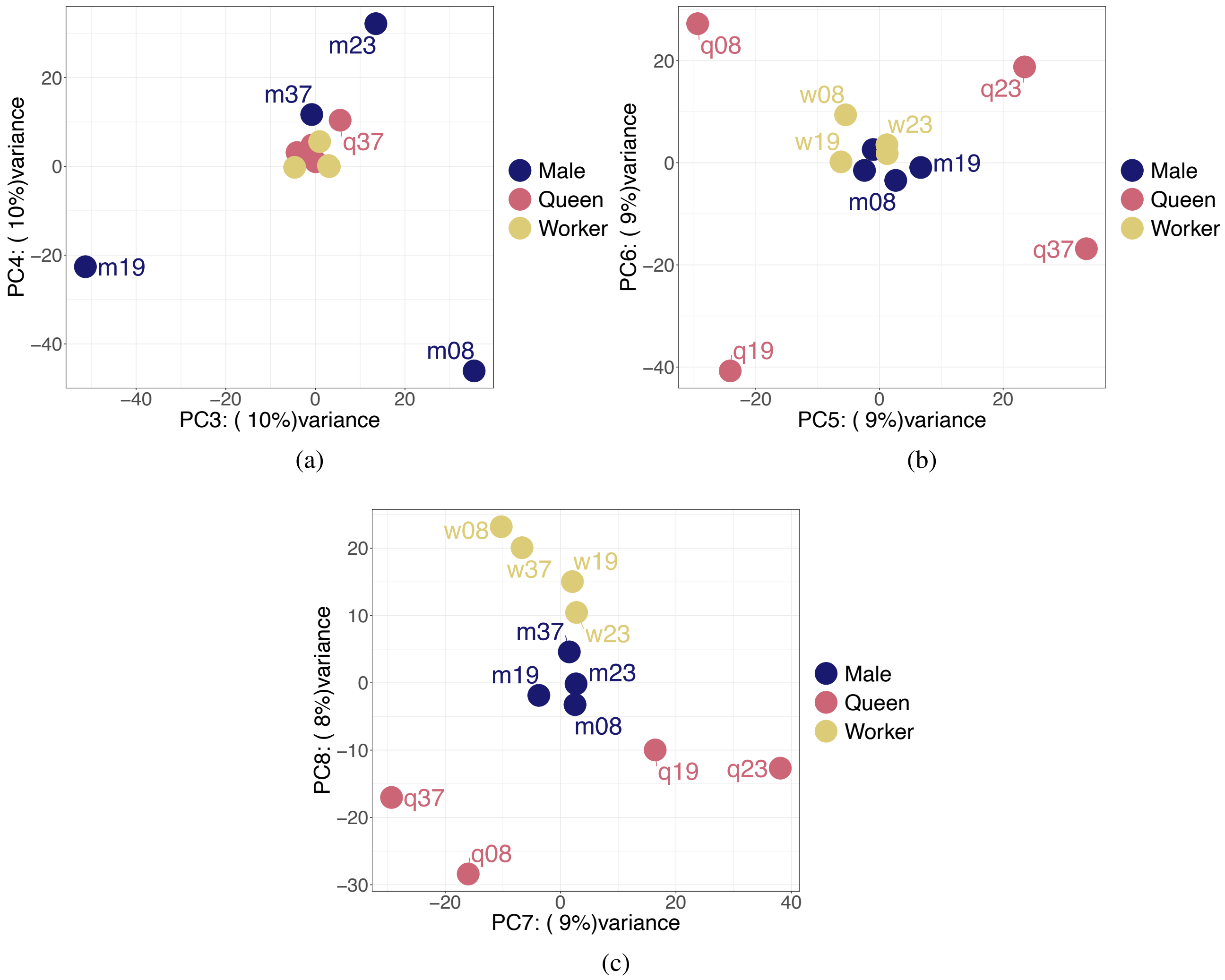

Figure S1: Additional PCA plots showing principle components 3-4 (a), 5-6 (b) and 7-8 (c).

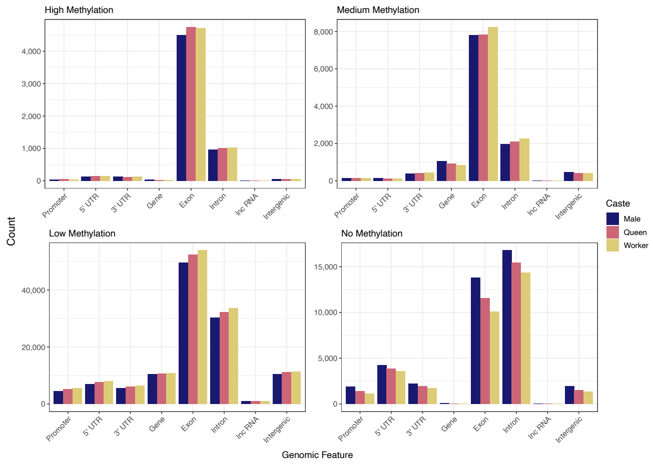

(a)

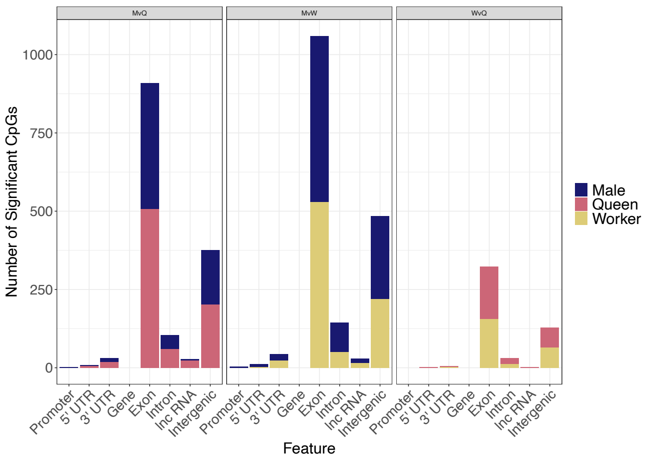

(b)

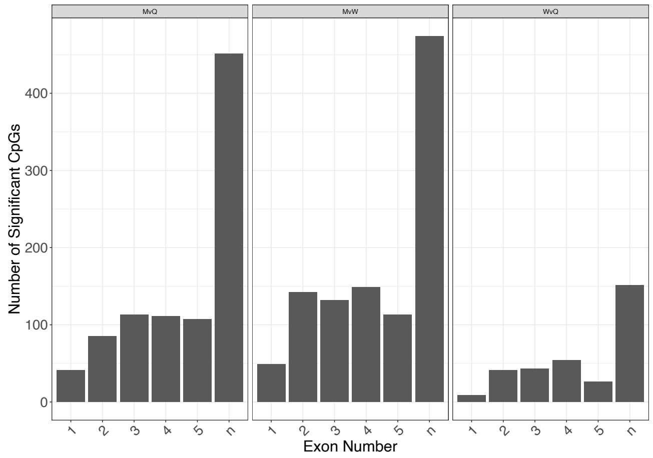

(c)

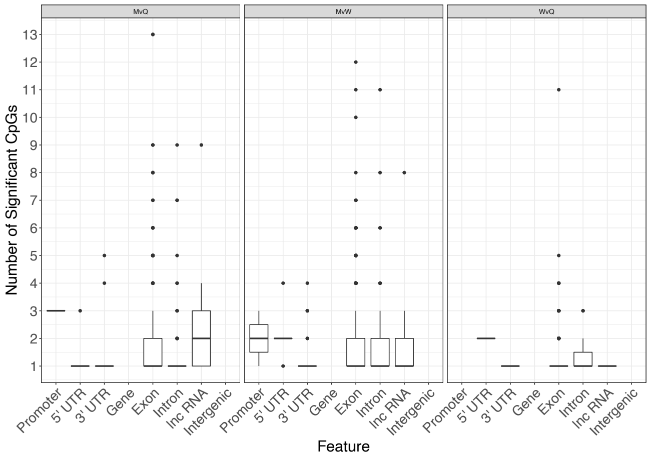

(d)

Figure S2: (a) Bar plots of the total number of genomic features categorised by weighted methylation level for sexes and castes. High methylation is a weighted methylation level >0.7, medium is >0.3-0.7, low is >0-0.3 and no methylation is equal to zero. (b) Staked bar chart showing the genomic location of the differentially methylated CpGs sites per comparison, coloured by the hypermethylated caste. (c) Barplot of the number of significant CpGs in the first five exons, with ’n’ representing exons six on-wards. (d) Boxplots of the number of significant CpGs per feature, per comparison, each dot represents an outlier.

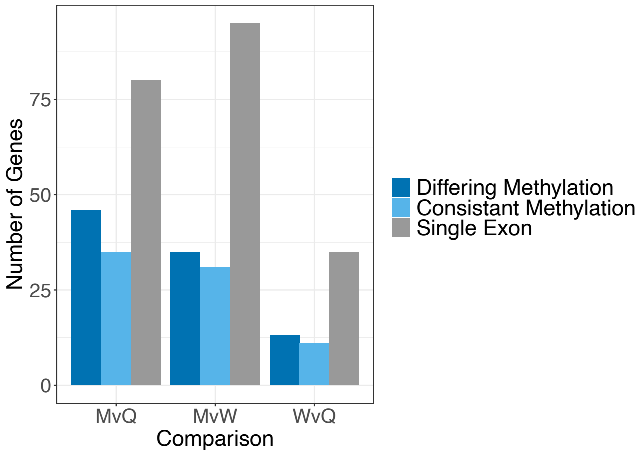

Figure S3: Bar plot showing the number of genes per comparison which either have a single differentially methylated exon or have multiple differentially methylated exons. Those genes with multiple differentially methylated exons are split by showing either all exons with consistent methylation difference (i.e. always hypermethylated) or showing differing methylation differences (i.e. some hypermethylated and other hypomethylated). MvQ represents the male vs queen comparison, MvW represents the male vs worker comparison and WvQ represents the worker vs queen comparison.

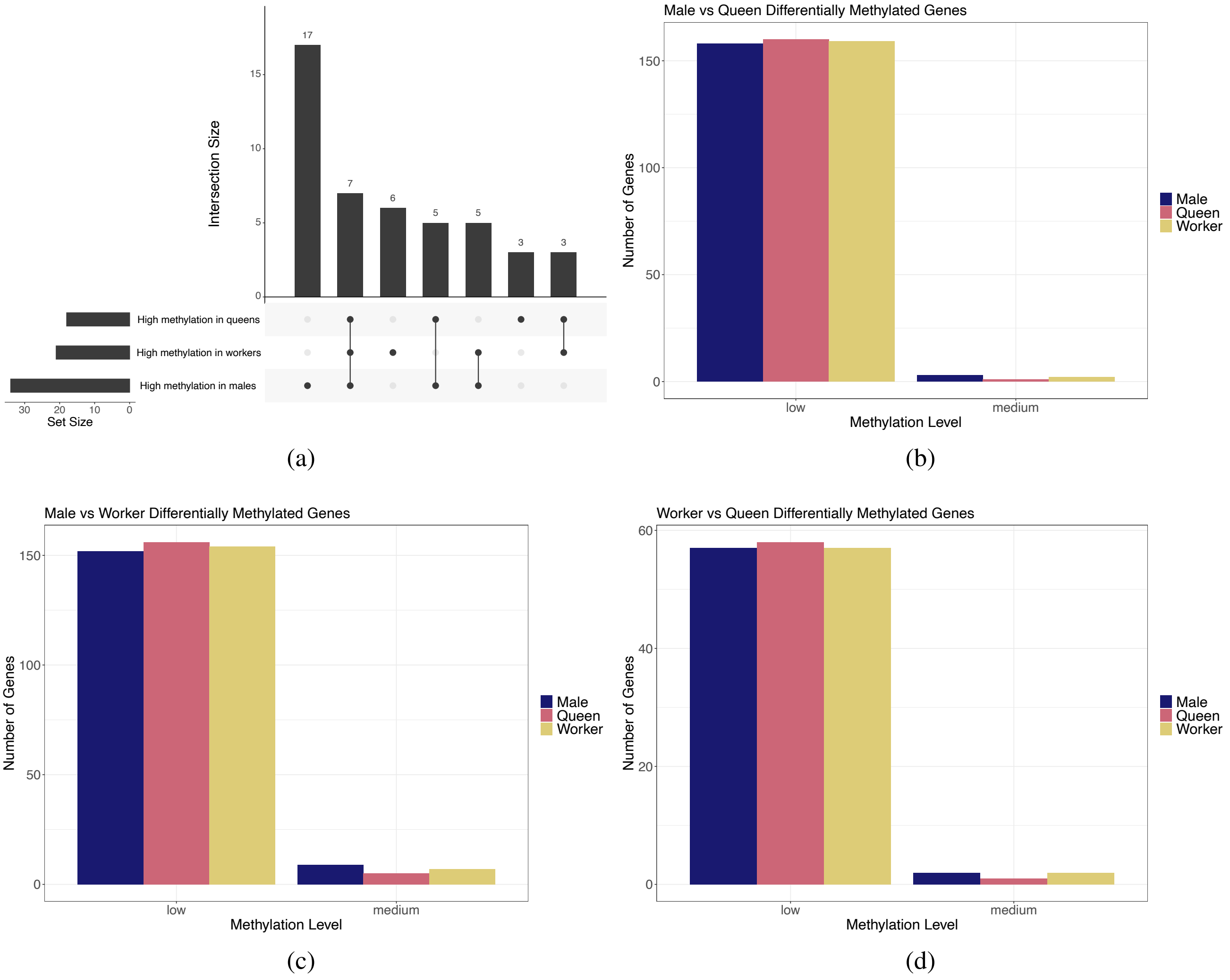

Figure S4: (a) UpSet plot showing the common genes which are highly methylated (>0.7 weighted methylation level) per sex/caste. The set size indicates the number of highly methylated genes, the intersection size shows how many of those are common between sets or unique, as indicated by connections in the bottom panel. (b - d) Overall methylation level of genes per caste which are differentially expressed between castes, for male vs queens, male vs workers and workers vs queens respectively. Low methylation refers to an overall weighted methylation >0 and <0.3, medium methylation refers to >= 0.3 and <0.7.

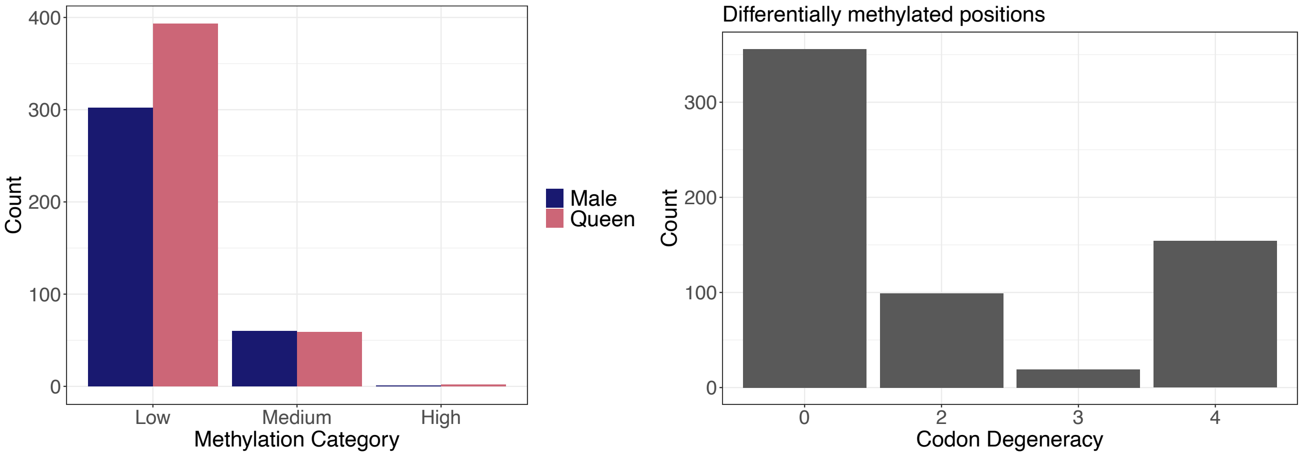

(a) (b)

Figure S5: (a) Bar plot showing the number of genes within each methylation category which contain at least one methylated zero-fold degenerate site. Genes with low methylation have a weighted methylation level >0 and <0.3, medium have >= 0.3 and <0.7 and high have >= 0.7. (b) Bar plot showing the degeneracy levels of the differentially methylated CpG sites found between queens and males. Of all differentially methylated CpG (n = 1034), around 63% are located in coding regions (n = 649).
